# The role of metabolic memory in diabetic kidney disease: identification of key genes and therapeutic targets

**DOI:** 10.3389/fphar.2024.1379821

**Published:** 2024-07-17

**Authors:** Tongyue Yang, Qi Feng, Mingwei Shao, Mengxing Pan, Feng Guo, Yi Song, Fengjuan Huang, Zhao Linlin, Jiao Wang, Lina Wu, Guijun Qin, Yanyan Zhao

**Affiliations:** ^1^ Division of Endocrinology, Department of Internal Medicine, The First Affiliated Hospital of Zhengzhou University, Zhengzhou, China; ^2^ Traditional Chinese Medicine Integrated Department of Nephrology, The First Affiliated Hospital of Zhengzhou University, Zhengzhou, China; ^3^ Research Institute of Nephrology, The First Affiliated Hospital of Zhengzhou University, Zhengzhou University, Zhengzhou, China

**Keywords:** diabetic kidney disease, metabolic memory, fibrosis, single-cell RNA sequencing, NDRG1

## Abstract

Diabetic kidney disease (DKD) is characterized by complex pathogenesis and poor prognosis; therefore, an exploration of novel etiological factors may be beneficial. Despite glycemic control, the persistence of transient hyperglycemia still induces vascular complications due to metabolic memory. However, its contribution to DKD remains unclear. Using single-cell RNA sequencing data from the Gene Expression Omnibus (GEO) database, we clustered 12 cell types and employed enrichment analysis and a cell‒cell communication network. Fibrosis, a characteristic of DKD, was found to be associated with metabolic memory. To further identify genes related to metabolic memory and fibrosis in DKD, we combined the above datasets from humans with a rat renal fibrosis model and mouse models of metabolic memory. After overlapping, NDRG1, NR4A1, KCNC4 and ZFP36 were selected. Pharmacology analysis and molecular docking revealed that pioglitazone and resveratrol were possible agents affecting these hub genes. Based on the *ex vivo* results, NDRG1 was selected for further study. Knockdown of NDRG1 reduced TGF-β expression in human kidney-2 cells (HK-2 cells). Compared to that in patients who had diabetes for more than 10 years but not DKD, NDRG1 expression in blood samples was upregulated in DKD patients. In summary, NDRG1 is a key gene involved in regulating fibrosis in DKD from a metabolic memory perspective. Bioinformatics analysis combined with experimental validation provided reliable evidence for identifying metabolic memory in DKD patients.

## Graphical Abstract

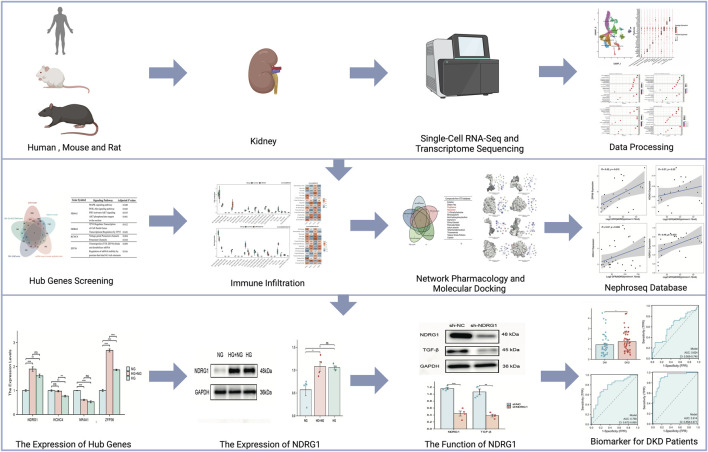

## 1 Introduction

Diabetic kidney disease (DKD) is the leading cause of end-stage kidney disease (ESKD) in the general population and accounts for approximately 50% of ESKD cases in the industrialized world (Collins et al., 2012; Novak et al., 2016). Although the sodium glucose co-transporter 2 inhibitor (SGLT2i) class has been shown to reduce the risk of kidney events in at-risk patients with type 2 diabetes mellitus (T2DM), efficient therapies arresting or even reversing DKD progression are lacking (Reidy et al., 2014; Wanner et al., 2016; Perkovic et al., 2019; Heerspink et al., 2020). Coincidentally, in 2003, the Diabetes Control and Complications Trial (DCCT) with further follow-up in the Epidemiology of Diabetes Interventions and Complications (EDIC) study (DCCT/EDIC) first proposed the concept of “metabolic memory.” These findings demonstrated that despite comparable HbA1c levels in both the intensive treatment group and the conventional treatment group, initial hyperglycemia remained a significant factor in elevating the risk of developing long-term diabetic complications, including DKD (Nathan et al., 2013). More recently, Al-Dabet et al. (2022) identified *p21* as the hub gene in hyperglycemic memory and demonstrated that the expression of *p21* was sustained at high levels regardless of glucose levels *in vitro* and *in vivo*, while inhibited *p21* expression could ameliorate high glucose-induced tubulointerstitial fibrosis in DKD.

Single-cell RNA sequencing (scRNA-seq) technology can amplify and sequence the transcriptome or genome at the single-cell level to detect the biological information of single cells in the fields of genomics, transcriptomics and proteomics, thus avoiding many limitations of traditional transcriptome sequencing (Jovic et al., 2022). Considering the diversity of cell types in DKD, an increasing number of studies have applied scRNA-seq to uncover the key genes involved in the onset and progression of DKD. Hirohama et al. (2023) recognized MMP7 as a diagnostic marker of kidney fibrosis through proteomics and scRNA-seq. Song et al. (2023) used scRNA-seq to screen RAC1 and demonstrated that RAC1 was involved in macrophage efferocytosis in DKD. However, at present, no study has screened key genes related to metabolic memory through scRNA-seq or explored the underlying mechanisms involved.

In this study, we used single-cell RNA sequencing (scRNA-seq) data derived from kidney samples of DKD patients, normal tissues adjacent to tumors, rat renal fibrosis models, and hyperglycemic memory mouse models to screen for crucial genes. Subsequently, we conducted cell cluster identification, enrichment analysis, and cell‒cell communication analyses on these selected datasets. By intersecting the datasets, we utilized immune infiltrate analysis, network pharmacology, molecular docking, and the Nephroseq database to elucidate the functional roles and clinical implications of the hub genes. Additionally, we employed quantitative real-time PCR (qRT‒PCR) and Western blotting to validate the expression levels of these genes in human kidney 2 (HK-2) cells. Furthermore, we assessed their levels in the blood of patients with diabetes for over 10 years but without DKD, as well as in DKD patients, using enzyme-linked immunosorbent assay (ELISA). Drawing from these findings, our objective was to scrutinize genes associated with metabolic memory and fibrosis in DKD, aiming to uncover potential molecular candidates that could illuminate novel avenues for the prevention of DKD.

## 2 Materials and methods

### 2.1 Data acquisition

**FIGURE 1:**
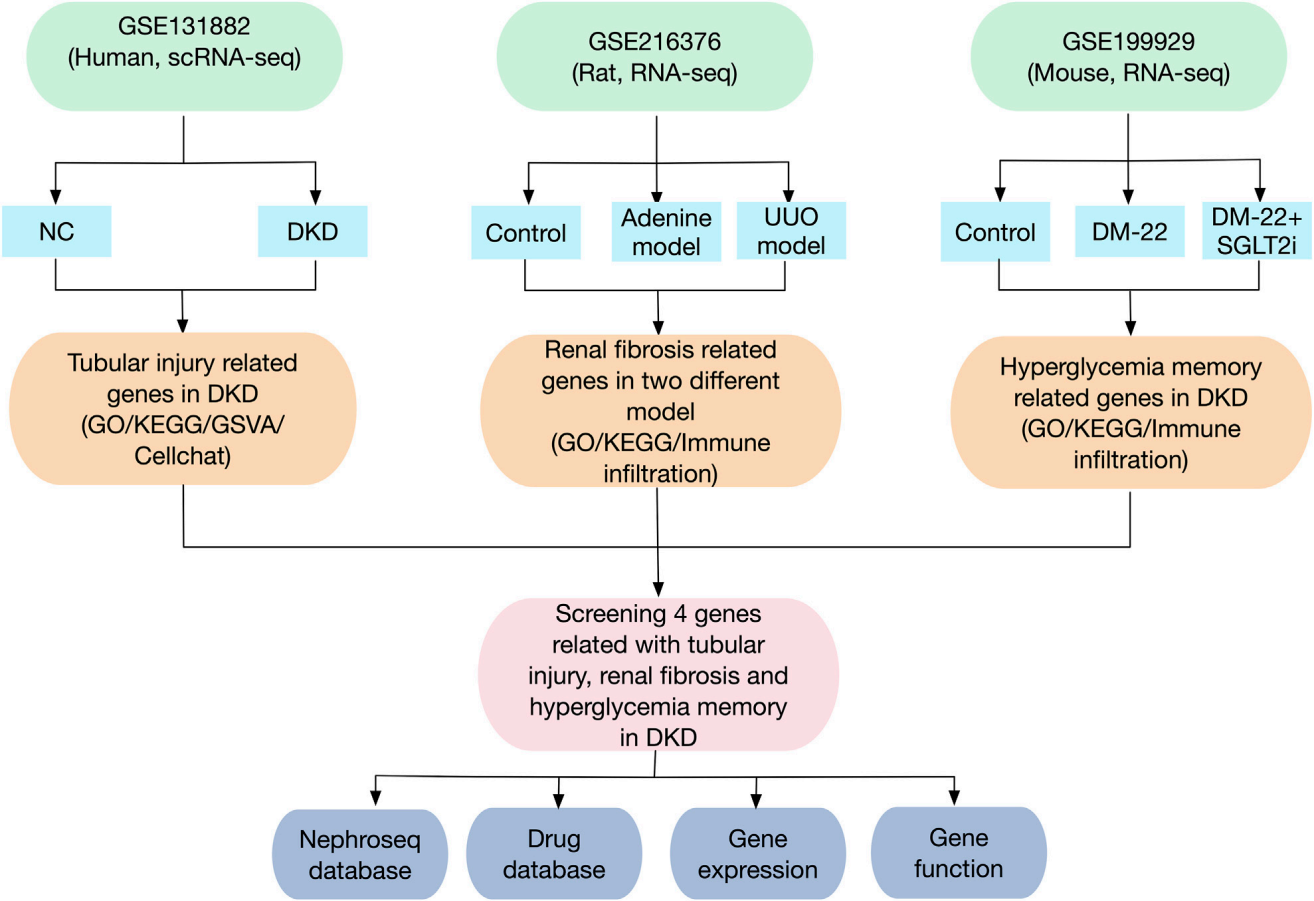
Flowchart of the research design and analysis process of the study.

The flowchart of this study is shown in Figure 1. The GEO database (https://www.ncbi.nlm.nih.gov/geo/) was used to obtain three gene expression datasets. The GSE131882 dataset comprises three samples of kidney tissue from DKD patients and three samples from normal tissue adjacent to tumors for scRNA-seq using the GPL24676 platform. The GSE216376 dataset is based on the GPL25947 platform and consists of three control models, three sham surgery models, three adenine-induced rat models and three UUO-induced rat models. The GSE199929 dataset (based on the GPL24247 platform) included data from three control models, two diabetic models and two diabetic mice treated with SGLT2i to reduce glucose levels.

### 2.2 scRNA-seq analysis

In this analysis, the exon, inex and intron sequences of each sample were taken as a single sample to obtain an expression matrix of 373,942 cells * 15,398 features. All cells that displayed nFeatures greater than 200 but less than 4,000 and a percentage of mitochondrial RNA less than 5% were included in the analysis, and a single-cell expression matrix with 2073 features was obtained for the 13,617 cells. The data were standardized by the NormalizeData function. After scale normalization, 2,000 highly variable genes (HVGs) were identified with the FindVariableFeatures function. ScaleData and RunPCA were used in turn to standardize the data, and PCA was performed (npcs = 50). Subsequently, the best PC value of this analysis was selected according to the elbow plot results. The best PC value was 15, and the cluster and tSNE values were obtained through FindNeighbors, FindClusters and RunTSNE. Clusters were then identified using FindClusters (resolution = 0.35). Cell type annotation was performed based on the top 10 HVGs in each cluster via manual checking and the CellMarkers dataset (http://xteam.xbio.top/CellMarker/).

### 2.3 Enrichment analysis

We used the R package “clusterProfiler” to perform Gene Ontology (GO) and Kyoto Encyclopedia of Genes and Genomes (KEGG) analyses. The packages “enrichGO” and “enrichKEGG” were also used. The “GSVA” Bioconductor package was subsequently used to implement gene set variation analysis (GSVA). An enrichment score was generated for each sample and pathway because of the use of a nonparametric unsupervised method that converted a traditional gene matrix (gene-by-sample) into a gene set by the sample matrix. The “heatmap” package was subsequently used to cluster the GSVA matrix, which was displayed as a heatmap. A *p*-value of < 0.05 was considered to indicate significant enrichment. In addition, we used the KOBAS 3.0 database (http://kobas.cbi.pku.edu.cn/) to determine the enrichment of KEGG pathways associated with NDRG1, NR4A1, KCNC4 and ZFP36.

### 2.4 Cell‒cell communication analysis

CellChat is a tool that uses network analysis, pattern recognition, and various learning techniques to quantitatively infer and analyze intercellular communication networks. The primary signaling inputs and outputs of cells, as well as how these cells and signals coordinate for functions, were predicted using a typical workflow. We then calculated the average ligand and receptor expression in a particular cell type by the mean value, which was determined by the proportion of cells between the DKD patients and the control group.

### 2.5 Immune cell infiltration analysis

RNA sequencing data from GSE199929 were analyzed using CIBERSORT, which was used to estimate the total immune infiltration in each sample and immune cell subset. The boxplots were visualized by the ggplot2 package, and the ggcorplot package was used for heatmap visualization.

### 2.6 Drug prediction and molecular docking of key target genes

The chemical interactions of the screened genes and disease information were acquired from the Comparative Toxicogenomics Database (CTD, http://ctdbase.org/). First, we created a Venn diagram using the R program VennDiagram to depict the intersection of the obtained drug targets of the 4 target genes and DKD. To investigate the interaction and binding activities of the compounds with the selected genes, molecular docking analysis was subsequently carried out. Protein 3D structures (https://www.rcsb.org/), including ZFP36 (PDB ID: 6NZL), KCNC4 (PDB ID: 1B4G), NR4A1 (PDB ID: 2QW4), and NDRG1 (PDB ID: 6ZMM), were collected from the Protein Data Bank database (PDB database). The molecular structures of pioglitazone and resveratrol were retrieved from the PubChem Compound Database (https://pubchem.ncbi.nlm.nih.gov/). After the legends were energy minimized and the hydrogen atoms of the receptors were removed, the molecular operating environment (MOE) was used for molecular modeling and ligand binding interactions.

### 2.7 Clinical correlation validation

Spearman rank correlation coefficient analysis was used to validate the association between the hub genes and clinical manifestations of DKD using the Nephroseq v5 online database (http://v5.nephroseq.org/), an integrated data-mining platform for gene expression datasets of kidney illnesses. A *p*-value of 0.05 indicated statistical significance.

### 2.8 Cell lines and cell culture

HK-2 cells were obtained from the Chinese Academy of Sciences (Shanghai, China) and cultured at 37°C in Dulbecco’s modified Eagle’s medium (HyClone, UT) supplemented with 10% fetal bovine serum (FBS; Gibco, CA), 2 mM glutamine, 100 U/mL penicillin, and 100 g/mL streptomycin. The cells were cultured in continuous normal (NG, 5.6 mM) glucose for 48 h, continuous high (HG, 30 mM) glucose for 48 h, or high glucose for 24 h followed by normal glucose for 24 h. The experimental scheme of the *in vitro* experiments is shown in Supplementary Figure S1. The *in vitro* experiments were repeated at least three independent times.

### 2.9 Knockdown of NDRG1

HK-2 cells were transfected with short hairpin RNA (shRNA) specific for NDRG1 (HANBIO, Shanghai, China) or with nontargeting shRNA (HANBIO, Shanghai, China) as a negative control. The transfection time was 24 h, and the multiplicity of infection (MOI) was 70. The sequence of the shRNA used was CCT​GGA​GTC​CTT​CAA​CAG​TTT. The above experiments were carried out following the instructions supplied by the manufacturer.

### 2.10 qRT‒PCR

Total RNA was extracted from HK-2 cells using TRIzol reagent (Invitrogen, United States) according to the manufacturer’s instructions. A Transcriptor First Strand cDNA Synthesis Kit (HiScript III All-in-one RT SuperMix Perfect for qRT‒PCR, Vazyme) was used to synthesize cDNA from mRNA using OligodT primers. To avoid deterioration, an RNase inhibitor was utilized. Taq Pro Universal SYBR qRT‒PCR Master Mix (Vazyme) and specific primer sets (Supplementary Table S1) were used for amplification. The expression of β-actin was used to normalize mRNA expression levels.

### 2.11 Western blot

HK‐2 cells were lysed in RIPA buffer (Cat# P0013B; Beyotime Biotechnology, China) with a protein inhibitor cocktail using the following primary antibodies: anti‐NDRG1 (Cell Signaling Technology–#9485), anti‐TGF-β (Abcam, ab215715) and anti‐GAPDH (Proteintech 10494-1-AP). Western blotting was performed after the sections were incubated with a horseradish peroxidase‐conjugated anti‐rabbit secondary antibody (D110011; Sangon Biotech, Shanghai, China).

### 2.12 Mouse model

C57BL/6J mice were obtained from Beijing Charles River (Beijing, China). Six-week-old male C57BL/6J mice were kept in a room at a temperature of 22°C–25°C with a 12-h light/dark cycle. After 2 weeks, streptozotocin (STZ; 50 mg/kg in 0.05 M citrate buffer, pH 4.5; Sigma‒Aldrich) was intraperitoneally injected into fasted mice for 12 h to establish a diabetic mouse model. The control mice were injected with citrate buffer. Diabetes was successfully induced by blood glucose levels greater than 16.7 mmol/L according to three consecutive tests when the fasting blood glucose level was tested 72 h after STZ injection. For a total of 16 weeks, body weight and blood glucose levels were monitored every 4 weeks.

### 2.13 Patients and controls

The participants included 43 patients with T2DM for more than 10 years but without DKD and 51 patients with DKD. Patients were recruited from the hospitalized patients of the First Affiliated Hospital of Zhengzhou University. The study was not a sex-specific human study. As a result, the sex distribution of the participants was not given special consideration. The sex of the participants was self-reported. The inclusion criteria were shown as follows: 1) Patients aged 20–80 years who have been diagnosed definitively diagnosed with T2DM. 2) DKD group: Patients who received a discharge diagnosis of DKD from the First Affiliated Hospital of Zhengzhou University, fulfilling the criteria of urinary albumin/creatinine ratio (UACR) > 30 mg/g and are accompanied by diabetic retinopathy (DR). 3) In the T2DM group: Patients with a history of diabetes for more than 10 years and a UACR < 30 mg/g were included. The exclusion criteria were shown as follows: 1) Patients who were diagnosed with chronic kidney disease caused by other diseases or triggers. 2) Patients who have been diagnosed with other diseases that lead to increased urinary protein. 3) Patients who were diagnosed with severe liver injury or malignancy were excluded. 4) Patients who have been diagnosed with other diseases that lead to increased urinary protein. All DKD patients were also diagnosed with diabetic retinopathy. The variables included age, sex, body mass index (BMI), systolic blood pressure (SBP), diastolic blood pressure (DBP), HbA1c (Hemoglobin A1c), fasting plasma glucose (FPG), serum creatinine (SCr), blood urea nitrogen (BUN), estimated glomerular filtration rate (eGFR), UACR, 24-h urinary protein quantity (24hUTP), duration of diabetes, and diagnosis of DR and hypertension. eGFR was calculated by the Modification of Diet in Renal Disease (MDRD) formula. The study was approved by the Ethics Committee of the First Affiliated Hospital of Zhengzhou University (Approval number: 2019-KY-228). All participants provided written informed consent in accordance with the Declaration of Helsinki.

### 2.14 ELISA

NDRG1 ELISA kits (ABclonal, Boston, United States) were used to analyze NDRG1 in the supernatant, and the procedure was performed exactly as directed by the manufacturer.

### 2.15 Statistical analysis

Comparisons between two and multiple groups were performed using independent t tests and one-way analysis of variance (ANOVA), respectively. R software (version 4.2.1) was used for statistical analysis. A *p*-value less than 0.05 was considered to indicate statistical significance.

## 3 Results

### 3.1 Preprocessing and cell cluster identification of scRNA-seq data

**FIGURE 2:**
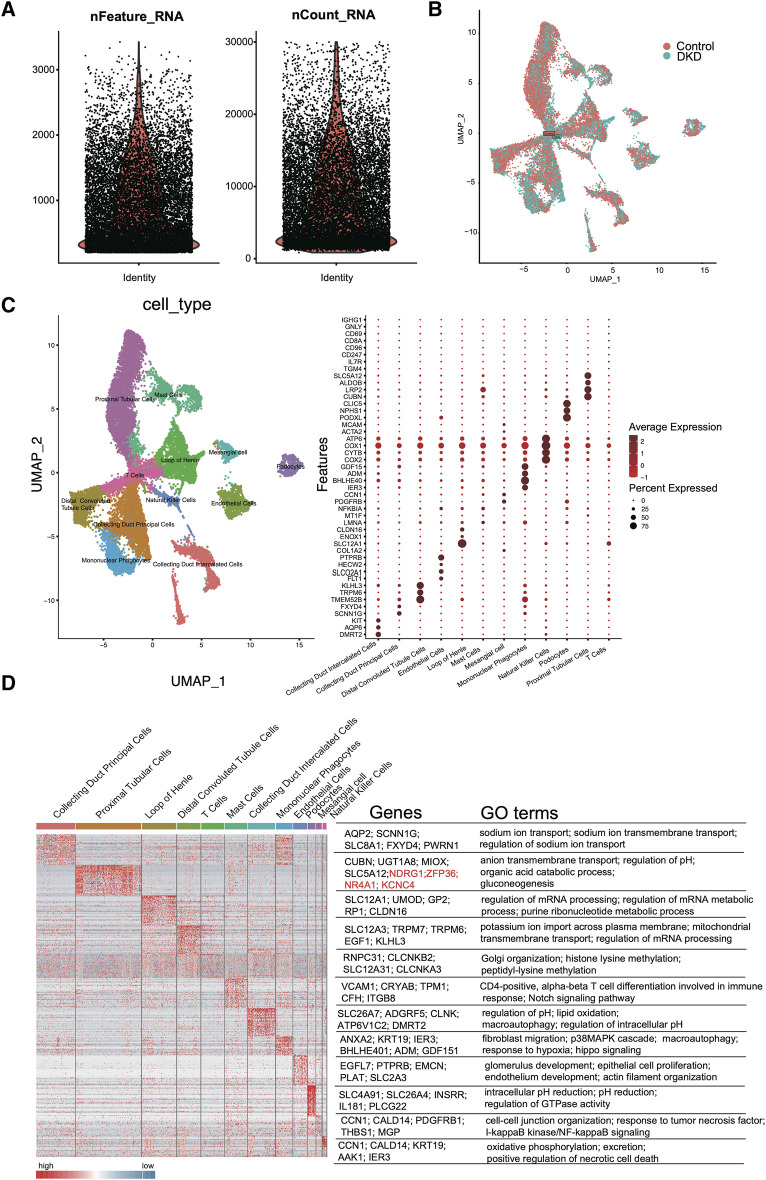
scRNA-seq quality control and analysis. **(A)** The number of unique RNA features (nFeatures) detected in each cell (left panel) and the total number of UMIs within a cell across all cells (nCounts) (right panel). **(B)** Uniform manifold approximation and projection (UMAP) for DKD and control samples. **(C)** Heatmap of DEGs in each cluster (left panel). The representative marker genes (middle panel) and the top GO terms (right panel) are shown. **(D)** Cell type UMAP representation (left panel) and dotplot of a selected set of cluster-specific genes (right panel).

As shown in Figure 2A, quality control of the aligned and counted reads was performed according to the *Methods* section. The data quality met the expectations. Next, we used t-distributed stochastic neighbor embedding (tSNE), principal component analysis (PCA), and uniform manifold approximation and projection (UMAP) for dimension reduction (Supplementary Figure S2; Figure 2B). The results suggested that UMAP may be a better choice for dimension reduction, and 12 major cell types were annotated by UMAP (Figure 2C). Different clusters were distinguished by cell markers; for example, proximal tubular cells were identified by CUBN, LRP2, SLC5A12 and TGM4; the loop of Henle was annotated by COL1A2, SLC12A1 and CLDN16; and distal convoluted tubule cells were identified by TMEM52B, TRPM6, and KLHL3 (Figure 2C). Among these cell types, tubular epithelial cells were obviously differentiated. To further verify the identified cell clusters, GO analysis was performed for each cluster. The representative genes and GO terms were shown in Figure 2D. For example, there were a large number of genes with high expression in collecting duct principal cells. The AQP2, SCNN1G, SLC8A1, FXYD4 and PWRN1 were selected as the representative genes, and the functions of these genes included sodium ion transport, sodium ion transmembrane transport and regulation of sodium ion transport, which were all classic functions of collecting duct principal cells.

### 3.2 Distribution and enrichment of scRNA-seq data

**FIGURE 3:**
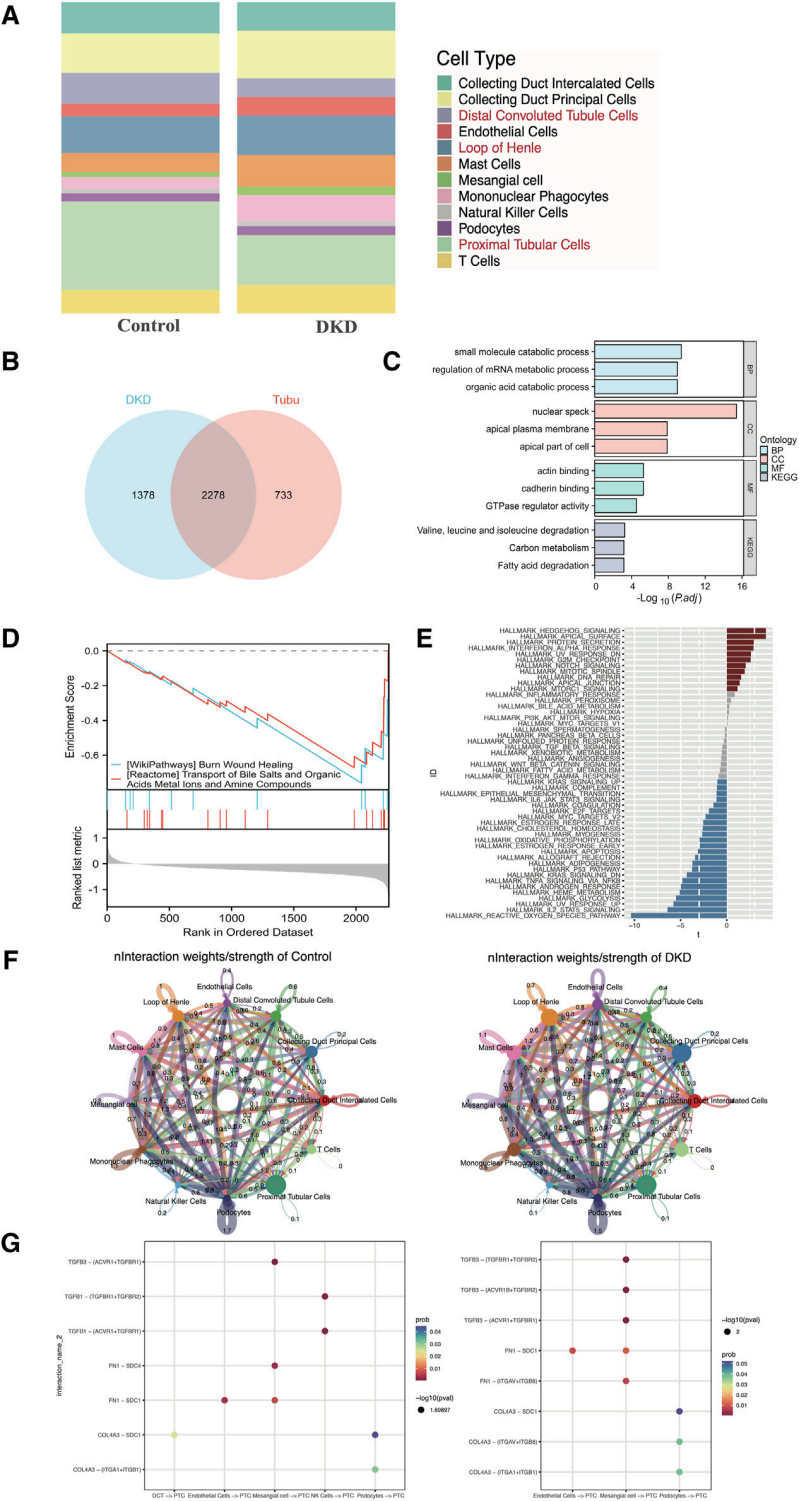
Distribution, selection, enrichment, and cell‒cell communication in GSE131882. **(A)** Stacked bar chart of the percentage of the cell types in DKD patients and control subjects. **(B)** Venn diagram showing the genes specifically expressed in tubular epithelial cells in DKD patients. **(C)** GO term and KEGG pathway enrichment analysis of the overlapping hub genes. **(D)** GSEA of the overlapping hub genes. **(E)** Differential pathway enrichment between the tubular epithelial cells of DKD patients and controls. **(F)** Interaction weights of tubular epithelial cells from DKD and control samples. **(G)** The intracellular ligand‒receptor signaling network in kidney cells from the DKD and control groups. The communication probability indicates the strength of the ligand‒receptor connection, and the P-value indicates the number of enriched genes. PCT, proximal tubular cell; DCT, distal convoluted tubulin; NK cells, natural killer cells.

As shown in Figure 3A, the percentage of tubular epithelial cells, including distal convoluted tubule cells, loop of Henle cells and proximal tubular cells, accounted for the largest proportion of cells, which was consistent with the findings of prior studies. In addition, as CREDENCE (Perkovic et al., 2019), EMPA-REG (Wanner et al., 2016) and DAPA-CKD (Heerspink et al., 2020) were carried out, and tubular epithelial cells had gained increased amounts of attention. Thus, we selected tubular epithelial cells as the research objects. After differential gene expression analysis, 3,011 DEGs were found to be specifically expressed in tubular epithelial cells, and 3,656 DEGs were found to be specifically expressed in DKD samples. Subsequently, 2,278 DEGs were identified (Figure 3B). To determine the possible biological functions of the overlapping DEGs, GO analysis, KEGG pathway analysis and GSEA analysis were carried out. The small molecule catabolic process, nuclear speck, actin binding and burn wound healing terms were enriched (Figures 3C, D). In addition, GSVA enrichment was performed to explore the differences between the DKD cluster and the control cluster in tubular epithelial cells, where the most common pathways, such as reactive oxygen species, IL-2, STAT5 and the UV response, were related to oxidative stress, inflammation, and DNA damage repair (Figure 3E).

### 3.3 Cell–cell crosstalk network of the scRNA-seq data

To clarify the underlying intercellular communications and cell state transitions in DKD, we analyzed the intercellular communication networks from the scRNA-seq data using the CellChat package. In the DKD sample, the number of interactions and interaction weights/strengths of all kinds of cells were greater than those in the control sample, especially for endothelial cells, mast cells and collecting duct principal cells (Figure 3F). In DKD, tubular epithelial cells may be stimulated by various factors, such as hyperglycemia, proteinuria, and oxidative stress; undergo phenotypic changes; and transform into fibroblasts that over synthesize the extracellular matrix, resulting in tubular interstitial fibrosis, which is one of the principal reasons leading to kidney function decline (Zhang et al., 2021; Zheng et al., 2021). To explore intercellular communication between tubular epithelial cells and other cells, we selected TGF-β, FN 1 and collagen as representative signaling pathways to identify receptor–ligand pairs (Figure 3G). In both the DKD and control groups, the expression of TGF-β signaling factors, such as TGFB3-TGFF3R1, increased from mesangial cells to proximal tubular epithelial cells. Collagen signaling molecules, such as COL4A3 − (ITGAV + ITGB8), were increased in DKD samples from podocytes to proximal tubular epithelial cells. However, these findings still needed to be experimentally confirmed.

### 3.4 Functional analysis of DEGs in mouse models of metabolic memory and rat models of renal fibrosis

**FIGURE 4:**
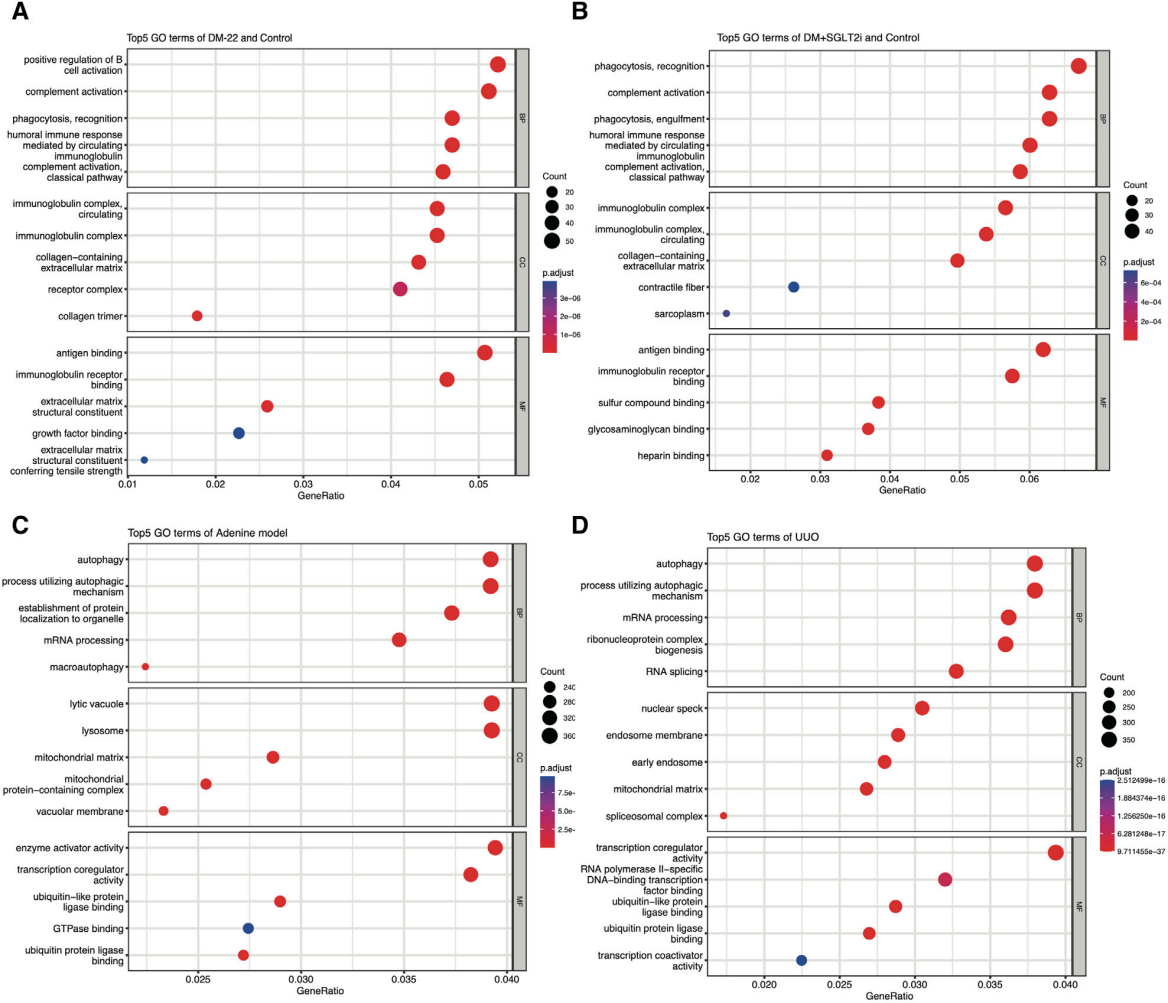
Enrichment analysis of GSE199929 and GSE216376. **(A)** Dotplot of the GO analysis of DEGs in nondiabetic mice (control) and diabetic mice (DM-22 model). **(B)** Dotplot of the GO analysis of DEGs in nondiabetic mice (control) and diabetic mice treated with SGLT2i to reduce glucose levels (DM + SGLT2i). **(C)** Dotplot of the GO analysis results for the adenine model. **(D)** Dotplot of the GO analysis results for the UUO model.

Al-Dabet et al. (2022) explored metabolic memory in tubular epithelial cells in DKD, but existing studies were still lacking. Thus, we used the GSE199929 dataset (Al-Dabet et al., 2022). They designed a nondiabetic mouse model (control), a diabetic mouse model without intervention to reduce blood glucose levels (DM-22) and a mouse model of hyperglycemia reversal by sodium-glucose cotransporter 2 inhibitors (SGLT2i, dapagliflozin) (DM-22+SGLT2i) to identify hub genes related to metabolic memory. As shown in Figures 4A, B, even though hyperglycemia was reversed, “phagocytosis,” “complement activation” and terms related to the immune response were still enriched, indicating that metabolic memory was present and might be associated with these phenotypes. Their research also indicated that metabolic memory was related to fibrosis, which is characteristic of progressive chronic kidney diseases of any etiology, including DKD, and eventually led to kidney failure (Hung et al., 2021). Therefore, we selected GSE216376, which consisted of two classic renal fibrosis rat models (adenine and UUO), to identify fibrosis-related DEGs. As shown in Figures 4C, D, the GO terms “autophagy,” “mRNA processing” and “ubiquitin-like protein ligase binding” were quite similar, suggesting that these two fibrosis models were in good agreement.

### 3.5 Identification and verification of hub genes related to fibrosis and metabolic memory in DKD

**FIGURE 5:**
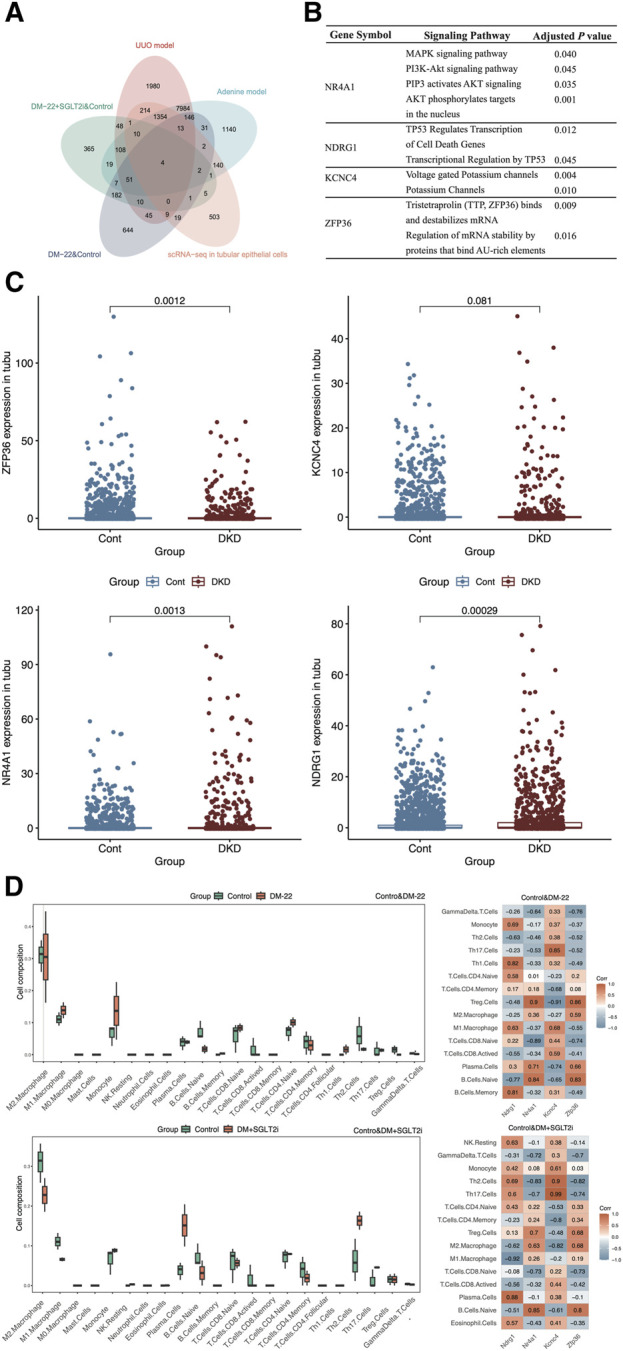
Screening hub genes. **(A)** The Venn diagram shows the overlapping genes obtained from five databases. **(B)** Enrichment analysis of ZFP36, KCNC4, NR4A1 and NDRG1. **(C)** Box plots of ZFP36, KCNC4, NR4A1 and NDRG1 expression levels in tubular epithelial cells from DKD and control samples. **(D)** Immune cell proportions and correlations between hub genes and infiltrating immune cells in control mice, diabetic mice (DM-22) and diabetic mice treated with SGLT2i to reduce glucose levels (DM + SGLT2i). *p < 0.05 and **p < 0.01 vs. the control group. ns, no significance.

After overlapping the different species datasets from multiple angles, NR4A1, NDRG1, KCNC4 and ZFP36 were screened (Figure 5A). To identify the functions of these key genes, we enriched four genes using KOBAS software (Figure 5B). Among these genes, NR4A1 was related to the MAPK and AKT signaling pathways, NDRG1 was associated with TP53-mediated transcription regulation, KCNC4 was annotated with potassium channels, and ZFP36 may regulate mRNA stability. The expression levels of NR4A1, NDRG1, KCNC4 and ZFP36 were again verified in tubular epithelial cells via the use of scRNA-seq data (Figure 5C). These findings provided clues for further study.

### 3.6 Analysis of the correlation between the hub genes and immune cell infiltration

As indicated by the results of the functional analysis of the metabolic memory models (Figures 4C, D), hyperglycemic memory might be associated with the immune response. Hence, we further explored the interactions between hub genes (NDRG1, NR4A1, KCNC4, and ZFP36) and immune cells (Figure 5D). M1 macrophages and monocytes were much more abundant in diabetic mice than in control mice, which indicated more pronounced inflammatory injury. After SGLT2i-mediated effects on glucose levels, the percentages of macrophages, monocytes, Th2 cells and Tregs changed, indicating immunosuppression, while the proportions of naive lymphocytes, including naive B cells and T cells, did not improve significantly. According to the heatmap, the expression of these hub genes could not be completely reversed after SGLT2i therapy; for example, the correlation between ZFP36 and naive B cells in the diabetic and control groups was 0.83, while that between the SGLT2i treatment group and the control group was 0.8. Thus, hyperglycemic memory might be correlated with the immune microenvironment in DKD.

### 3.7 Potential drug prediction and molecular docking of the hub genes

**FIGURE 6:**
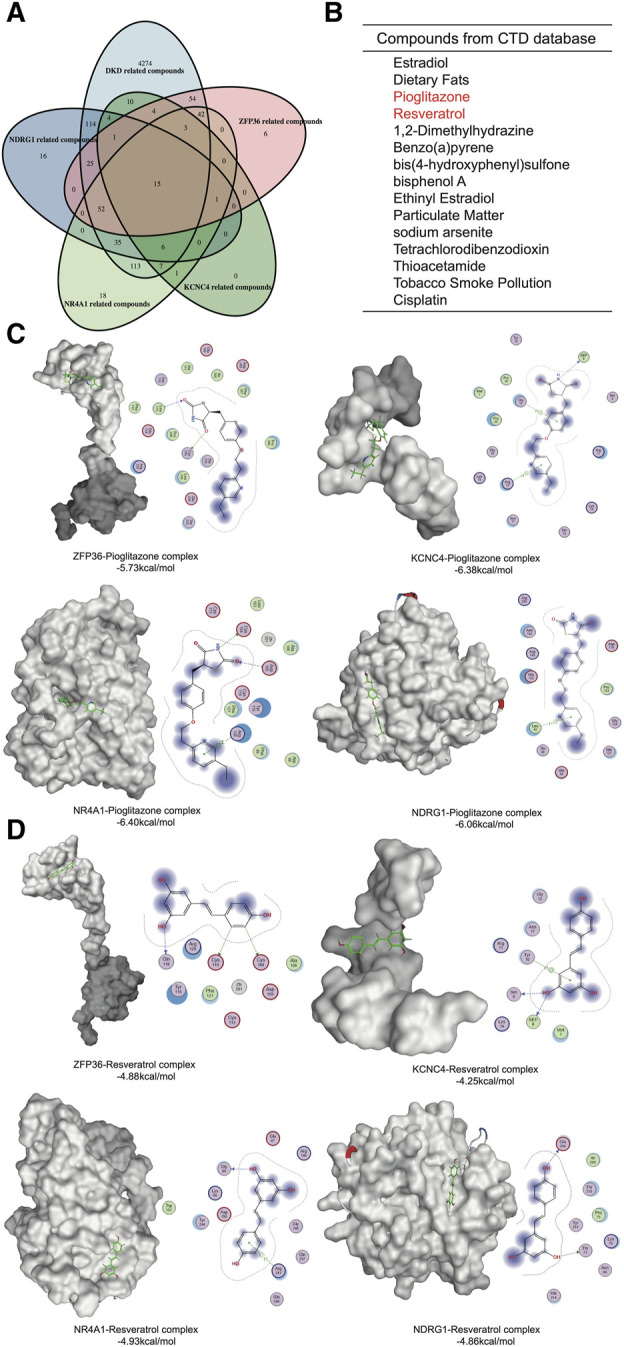
Network pharmacology and molecular docking. **(A)** Venn diagram of the gene-related compounds and DKD-related compounds. **(B)** The results of the Venn diagram. **(C)** Molecular docking of pioglitazone and the hub proteins. **(D)** Molecular docking of Resveratrol and the hub proteins.

To identify potential drugs against these hub genes, we screened components from the CTD platform (Figures 6A, B). Among the 15 identified compounds, pioglitazone and resveratrol had well-defined kidney protection effects and were selected for molecular docking analysis (Ho et al., 2022; Gu et al., 2022). The binding free energy represented the intermolecular binding ability. The 2D molecular model included detailed information about the ligand–receptor interactions, such as hydrogen bonding, hydrophobic bonding and carbon–hydrogen bonding. A binding free energy <0 kcal/mol indicated that the protein–ligand complex can dock in a natural state, an affinity energy < −1.2 kcal/mol indicated good docking, and a binding energy ≤ −5 kcal/mol indicated strong docking. The affinities of pioglitazone for all four proteins were less than −5 kcal/mol (Figure 6C). Although the binding force of resveratrol was much greater than that of pioglitazone, the affinity of resveratrol was quite close to −5 kcal/mol (Figure 6D). Therefore, pioglitazone and resveratrol might be effective against these key genes and metabolic memory.

### 3.8 Validation of the clinical significance of the hub genes

**FIGURE 7:**
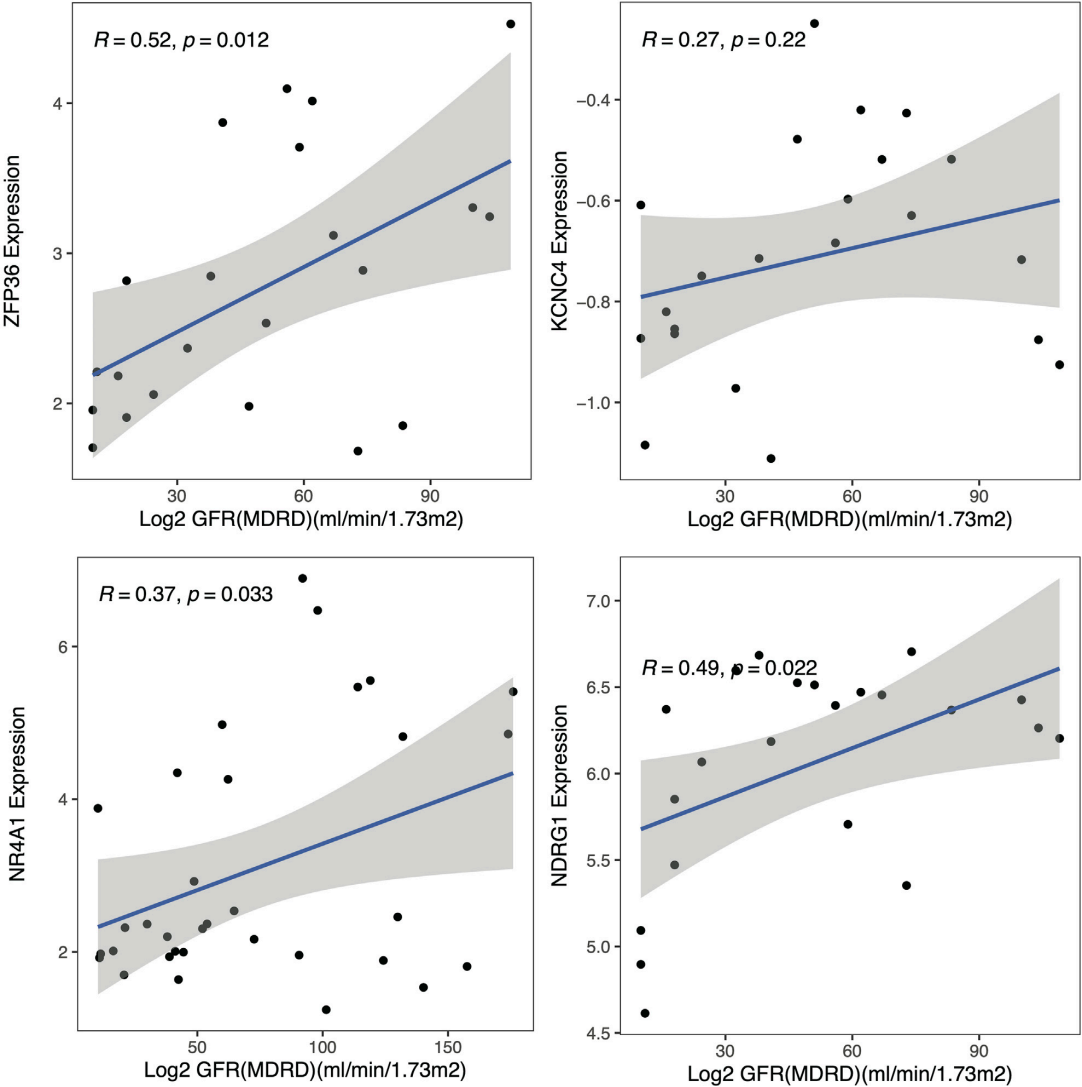
Clinical analysis. Correlation analysis of the hub genes with the glomerular filtration rate (GFR).

To explore the clinical significance of the identified hub genes, correlation analysis between the expression of these genes and the eGFR was conducted with the Nephroseq v5 online tool (Figure 7). All these genes were positively correlated with the GFR, while the *P* values of NDRG1, NR4A1 and ZFP36 were less than 0.05, and the associations were 0.49, 0.37 and 0.52, respectively.

### 3.9 NDRG1 is a metabolic memory- and fibrosis-related hub gene

**FIGURE 8:**
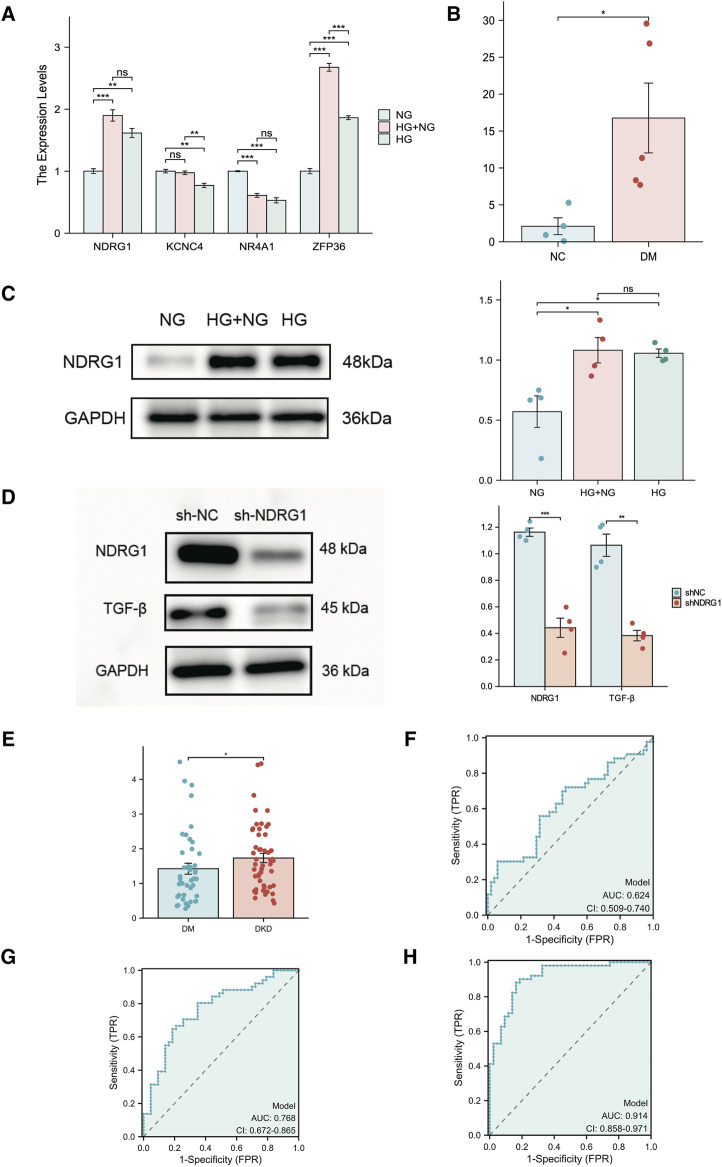
Experimental verification of hub genes related to fibrosis and metabolic memory in DKD patients. **(A)** The expression of NR4A1, NDRG1, KCNC4 and ZFP36 was analyzed via RT‒PCR in HK-2 cells (n = 3 per sample). **(B)** The expression of NDRG1 in kidney tissue from diabetic mice (DM) and nondiabetic mice (NC) was analyzed via RT‒PCR (n = 4 per group). **(C)** Western blotting was used to verify the expression of NDRG1 in HK-2 cells (n = 4 for each). **(D)** Western blotting was used to verify the knockdown efficiency of NDRG1 and to detect the protein expression level after TGF-β was knocked down by NDRG1 (n = 4 per group). **(E)** The expression of NDRG1 in the blood of DKD patients (n = 51) compared with that in the blood of DM patients without DKD for more than 10 years (n = 43). **(F)** ROC curve analysis of NDRG1 expression in the blood of DKD patients and DM patients without DKD for more than 10 years. **(G)** ROC curve analysis of NDRG1 expression combined with age, BMI and SBP. **(H)** ROC curve analysis of NDRG1 expression combined with age, BMI and SBP, Scr, BUN and eGFR. HK-2 cells: human kidney-2 cells; NG: control with continuous normal glucose (5.6 mM glucose) for 48 h; HG: control with continuous high glucose (30 mM glucose) for 48 h; HG + NG: high glucose (30 mM glucose) for 24 h followed by normal glucose (5.6 mM glucose) for 24 h *P < 0.05 or **P < 0.01 or ***P < 0.001.

To examine the expression of these hub genes, we cultured HK-2 cells *in vitro* in the presence of continuous normal glucose (NG) or high glucose (HG) for 24 h followed by HG (HG + NG) or continuous HG. The results indicated that the mRNA and protein levels of NDRG in HK-2 cells remained elevated, even after a further 24-h reduction in glucose concentration (Figures 8A, C). While ZFP36 expression increased under HG conditions, it sharply declined upon transitioning to NG conditions, failing to adhere to metabolic memory criteria. Conversely, NR4A1 expression, though in line with metabolic memory standards, was comparatively low. We also validated NDRG1 expression in diabetic mice with albuminuria, which indicated that NDRG1 was a causative gene in DKD (Figure 8B). Thus, we selected NDRG1 for further validation. Among the fibrosis-related pathways, the TGF-β signaling pathway is one of the most common pathways. In addition, Menezes et al. (2019) illustrated that NDRG1 could inhibit TGF-β signaling to enhance membrane E-cadherin expression in pancreatic cancer. Thus, we knocked down NDRG1 in HK-2 cells, and the results showed that NDRG1 reduced TGF-β expression in HK-2 cells cultured with HG (Figure 8D).

### 3.10 Diagnostic performance of NDRG1

To further clarify the potential diagnostic value of NDRG1, we collected and conducted a univariate analysis on the general information, main laboratory test results, comorbidities, and NDRG1 expression levels in blood samples from patients with T2DM for more than 10 years but without DKD (T2DM Group) and patients with DKD and DR (DKD Group) (Table 1). The results indicated that the NDRG1 expression level, diabetes duration, age, BMI, SBP, HbA1c, SCr, BUN, UACR, total 24-h urine protein, and the presence of DR in the DKD Group were significantly different from those in the T2DM Group (*P* < 0.05). Since diabetes duration, DR, UACR, and total 24-h urine protein are inclusion criteria for the T2DM Group and DKD Group, these four indicators were not included in subsequent statistical analyses. Among the DKD patients, there were 24 individuals whose eGFR exceeded 100, yet all of them fulfilled the diagnostic criteria for DKD. DKD patients (n = 51) exhibited significantly greater NDRG1 expression than DM patients (Figure 8E). Although the area under the receiver operating characteristic (ROC) curve (AUC) for NDRG1 expression did not reach 0.7, NDRG1 expression combined with demographic and anthropometric indicators (age, BMI and SBP) was 0.768 (95% CI 0.672–0.865) (Figures 8F, G). When further combined with biochemical indicators (SCr, BUN and eGFR), the area under the ROC curve reached 0.914 (95% CI 0.858–0.971) (Figure 8H). The accuracy, sensitivity and specificity were 0.862, 0.882 and 0.837, respectively. Thus, an increase in the blood NDRG1 concentration might be a potential biomarker for DKD diagnosis.

**TABLE 1:** Characteristics of participants with T2DM and DKD.

Variables	Total (n = 94)	T2DM group (n = 43)	DKD group (n = 51)	*P-value*
NDRG1 (ng/mL)	1.37 (0.84, 2.03)	1.13 (0.65, 1.87)	1.56 (0.92, 2.4)	0.04
Duration of DM, (years)	12 (10, 18)	13 (10, 18)	10 (5, 17.5)	0.01
Gender, n (%)				0.96
Female	38 (40)	18 (42)	20 (39)	
Male	56 (60)	25 (58)	31 (61)	
Age (years)	59 (51.25, 66)	60 (55, 68)	58 (48, 65.5)	0.05
BMI, (kg/m^2^)	22.94 (21.93, 25.81)	23.68 (22.62, 26.81)	22.76 (20.5, 24.32)	0.03
SBP (mmHg)	130.98 ± 19.73	125.56 ± 18.06	135.55 ± 20.08	0.01
DBP (mmHg)	77.65 ± 14.2	74.74 ± 11.89	80.1 ± 15.59	0.06
Haemoglobin (g/L)	127.63 ± 21.96	131.58 ± 16.69	124.29 ± 25.27	0.10
HbA1c (%)	8.6 (7.3, 10.1)	7.7 (7.15, 9.4)	9.2 (7.95, 11.55)	0.01
FPG (mmol/L)	7.98 (5.54, 10.52)	7.54 (5.72, 9.05)	8.59 (5.28, 12.3)	0.27
SCr (umol/L)	70.5 (57.25, 84.75)	64 (55.5, 74.5)	75 (61.5, 103.5)	< 0.01
BUN (mmol/L)	6.94 (5.55, 8.28)	5.62 (4.95, 7.5)	7.05 (6.39, 9.51)	< 0.01
eGFR (ml/min/1.73m^2^)	108.75 (86.93, 133.4)	120.17 (99.72, 139.08)	97.32 (67.8, 126.5)	< 0.01
UACR (mg/g)	61.38 (5.23, 466.09)	4.9 (2.4, 7.25)	428.06 (134.93, 1,154.05)	< 0.01
24hUTP (g)	0.31 (0.21, 0.64)	0.21 (0.17, 0.26)	0.61 (0.4, 1.31)	< 0.01
DR, n (%)				< 0.01
No	24 (26)	24 (56)	0 (0)	
Yes	70 (74)	19 (44)	51 (100)	
Hypertension, n (%)				1
No	51 (54)	23 (53)	28 (55)	
Yes	43 (46)	20 (47)	23 (45)	

Continuous variables with a normal distribution are expressed as the mean ± SD, and nonnormal data are expressed as the median (interquartile range, IQR).

Abbreviations: DM, diabetes mellitus; DKD, diabetic kidney disease; BMI, body mass index; SBP, systolic blood pressure; DBP, diastolic blood pressure; HbA1c, hemoglobin A1c; FPG, fasting plasma glucose; SCr, serum creatinine; BUN, blood urea nitrogen; eGFR, estimated glomerular filtration rate; UACR, urinary albumin/creatinine ratio; 24hUTP, 24-h urinary protein quantity; DR, diabetic retinopathy.

## 4 Discussion

The pathogenesis of DKD is complex and involves classic mechanisms, such as inflammation (Rayego-Mateos et al., 2023) and oxidative stress (Park et al., 2023), as well as several emerging mechanisms, such as pyroptosis (Qu et al., 2022) and ferroptosis (Li et al., 2021). However, even though multiple hypoglycemic agents are currently available for improving the above mechanisms and reducing blood glucose levels, the initiation of high blood glucose-induced damage in the kidney is still ongoing (Zheng et al., 2021). This latent effect across conditions and time is associated with the occurrence and development of chronic diabetic complications termed “metabolic memory.” Recent studies have shown that metabolic memory plays an important role in the pathogenesis of DKD (Li et al., 2022; Al-Dabet et al., 2022). However, the specific mechanism of metabolic memory in DKD remains unclear. Hence, we combined scRNA-seq data from humans and high-throughput sequencing data from mice and rats to investigate the possible mechanisms of metabolic memory in tubular epithelial cell fibrosis in DKD. We also used network pharmacology and molecular docking to identify the potential regulatory effects of these metabolic memory-related genes. Our experimental validation revealed that NDRG1 was a metabolic memory-related gene that regulated TGF-β expression and was a potential biomarker for DKD diagnosis.

The concept of metabolic memory was first proposed in 2003; in recent years, numerous studies have started to explore the mechanisms behind this phenomenon. Li et al. (2022) reported that *Sirt7* cooperated with ELK1 to induce inflammation in endothelial cells despite the restoration of normoglycemia. Lizotte et al. (2016) also demonstrated that despite reduced blood glucose levels resulting from insulin treatment for the last 2 months, the expression of SHP-1 remained elevated in the podocytes of diabetic mice. The authors focused on the relationship between metabolic memory and glomerular injury in DKD patients. Although glomerular injury is essential for the progression of DKD, tubular epithelial cells have gained increasing attention in the clinical application of SGLT2is. Several studies have confirmed that tubular epithelial cell injury is the prime and important factor that impacts the progression of DKD (Wang et al., 2023; Shen et al., 2022). Growing evidence has demonstrated that tubular epithelial cells change and become fibrogenic in response to hyperglycemia-induced injury. This results in tubulointerstitial fibrosis, which is one of the most prevalent pathological features of DKD (Cui et al., 2022; Ji et al., 2023). Recent research has shown that methylation of the senescence-related gene *p21* regulates metabolic memory and fibrosis in tubular epithelial cells in DKD (Al-Dabet et al., 2022). Regrettably, studies on the role of metabolic memory in DKD, especially those focused on tubular epithelial cells, are relatively rare.

In our research, we evaluated the potential of pioglitazone and resveratrol as therapeutic agents against metabolic memory in patients with DKD. While pioglitazone is not conventionally prescribed for DKD treatment, it has demonstrated the ability to hinder renal fibrosis in diabetic mice (Gan et al., 2023). However, the precise therapeutic function of these compounds in addressing metabolic memory remains elusive and demands further exploration. Resveratrol, an antioxidant and kidney-beneficial natural nonflavonoid polyphenolic compound (Blahova et al., 2021; Sattarinezhad et al., 2019), is particularly noteworthy as oxidative stress is a pivotal mechanism underlying metabolic memory, particularly in DR (Lee et al., 2022; Wang et al., 2018; Voronova et al., 2017). Chen et al. (2023) utilized network pharmacology and molecular docking to identify potential target genes of resveratrol in DKD, yet our key genes were not encompassed in their study. Additionally, Xian et al. (2020) hypothesized that resveratrol mitigates oxidative stress induced by metabolic memory in mice, aligning with our findings. Regrettably, cytological experiments were not performed to determine the underlying molecular mechanisms involved.

N-myc downstream-regulated gene 1 (NDRG1) is a member of the NDRG family and is a highly conserved and widely expressed gene located on chromosome 8 at the 8q24.2 locus (Fang et al., 2014). The most studied functions of NDRG1 include tumor metastasis and hypoxia (Park et al., 2020; Joshi et al., 2022). Although NDRG1 has been identified in the mitochondrial inner membrane of proximal tubule cells in the kidney, where it is regulated by HIF, the current literature lacks studies exploring its expression and function in DKD (Lachat et al., 2002; Zhang et al., 2020). Previous studies have confirmed that mitochondrial dysfunction was closely related to fibrosis in DKD and metabolic memory in DR (Forbes and Thorburn, 2018; Wang et al., 2018). For instance, Keshava et al. (2023) demonstrated that reduced NDRG1 expression attenuated pleural fibrosis. In pancreatic cancer, NDRG1 suppressed TGF-β and NF-κB signaling, thereby enhancing membrane E-cadherin expression. Our research revealed that the mRNA level of NDRG1 in kidney tissue was positively related to the eGFR. To validate NDRG1 expression, we employed RT‒qPCR and Western blotting in HK-2 cells and diabetic mouse models. Our findings indicate that NDRG1 expression is upregulated by high glucose both *in vitro* and *in vivo*, with a particularly robust increase observed in HK-2 cells after 24 h of exposure to a high glucose culture solution. Moreover, knocking down NDRG1 in HK-2 cells suppressed TGF-β expression, consistent with previous studies. Furthermore, blood samples from DKD patients, but not DKD patients, displayed higher NDRG1 expression than did those from patients with T2DM for over 10 years. Considering demographic, anthropometric, and biochemical parameters, NDRG1 has emerged as a potential biomarker that could aid in distinguishing DKD patients, potentially without interfering with urine test results.

Our study has several strengths. First, we combined scRNA-seq data from humans with high-throughput sequencing data from a mouse model of hyperglycemia and two renal fibrosis models from rats to ensure the stability of NDRG1 in different species. Second, we focused on tubular epithelial cells and performed experimental validation. Third, we selected patients who had diabetes for more than 10 years but without DKD as the control group to ensure the accuracy of the test. Given that our research still needs mechanistic exploration, several potential limitations should be considered. First, although the expression level of NDRG1 was validated at the cellular level in this study, the expression level and underlying mechanisms of NDRG1 in DKD patients and animal models of metabolic memory remain to be further elucidated. Second, despite previous research indicating that resveratrol and pioglitazone can alleviate renal tubular epithelial fibrosis, the specific mechanisms by which they improve metabolic memory in DKD and renal tubular epithelial fibrosis still await experimental verification.

## 5 Conclusion

In summary, this study analyzed scRNA-seq and high-throughput sequencing data from multiple species and performed potential drug prediction and molecular docking analyses. After experimental validation, we identified NDRG1 as a potential hub gene of metabolic memory in DKD patients. Additionally, NDRG1 may reduce fibrosis in tubular epithelial cells through the TGF-β pathway and may also be a potential biomarker for DKD patients, shedding light on basic and drug research on DKD.

## Data Availability

The datasets presented in this study can be found in online repositories. The names of the repository/repositories and accession number(s) can be found in the article/Supplementary Material.
