# Supplementary material for: The role of metabolic memory in diabetic kidney disease: identification of key genes and therapeutic targets
Source: Front Pharmacol. 2024 Jul 17;15:1379821. doi: 10.3389/fphar.2024.1379821 (PMC11292736; doi:10.3389/fphar.2024.1379821)
Supplement: Supplementary file 1 [file DataSheet1.docx]

**SUPPLEMENTAL MATERIALS**

**the Role of Metabolic Memory in Diabetic Kidney Disease: Identification of Key Genes and Therapeutic Targets**

Tongyue Yang^1#^, Feng Qi^2,3#^, Mingwei Shao^1^, Mengxing Pan^1^, Feng Guo^1^, Yi Song^1^, Fengjuan Huang^1^, Zhao Linlin^1^, Jiao Wang^1^, Lina Wu^1^, Guijun Qin^1^, Yanyan Zhao^1^*

^#^ Shared first authorship

^1^Division of Endocrinology, Department of Internal Medicine, The First Affiliated Hospital of Zhengzhou University, Zhengzhou 450052, China.

^2^ Traditional Chinese Medicine Integrated Department of Nephrology, the First Affiliated Hospital of Zhengzhou University, Zhengzhou 450052, China.

^3^Research Institute of Nephrology, Zhengzhou University, the First Afﬁliated Hospital of Zhengzhou University, Zhengzhou 450052, P. R. China.

## * Corresponding author

Yanyan Zhao, MD, PhD, Division of Endocrinology, Department of Internal Medicine, The First Affiliated Hospital of Zhengzhou University, Zhengzhou 450052, China. Email: [fcczhaoyy1@zzu.edu.cn](mailto:fcczhaoyy1@zzu.edu.cn)

**Table of Contents**

Supplementary Figure 12

Supplementary Table 13

Supplementary Figure 24

**Supplementary Figure 1. Experimental scheme of in vitro experiments with HK-2 cells**

**
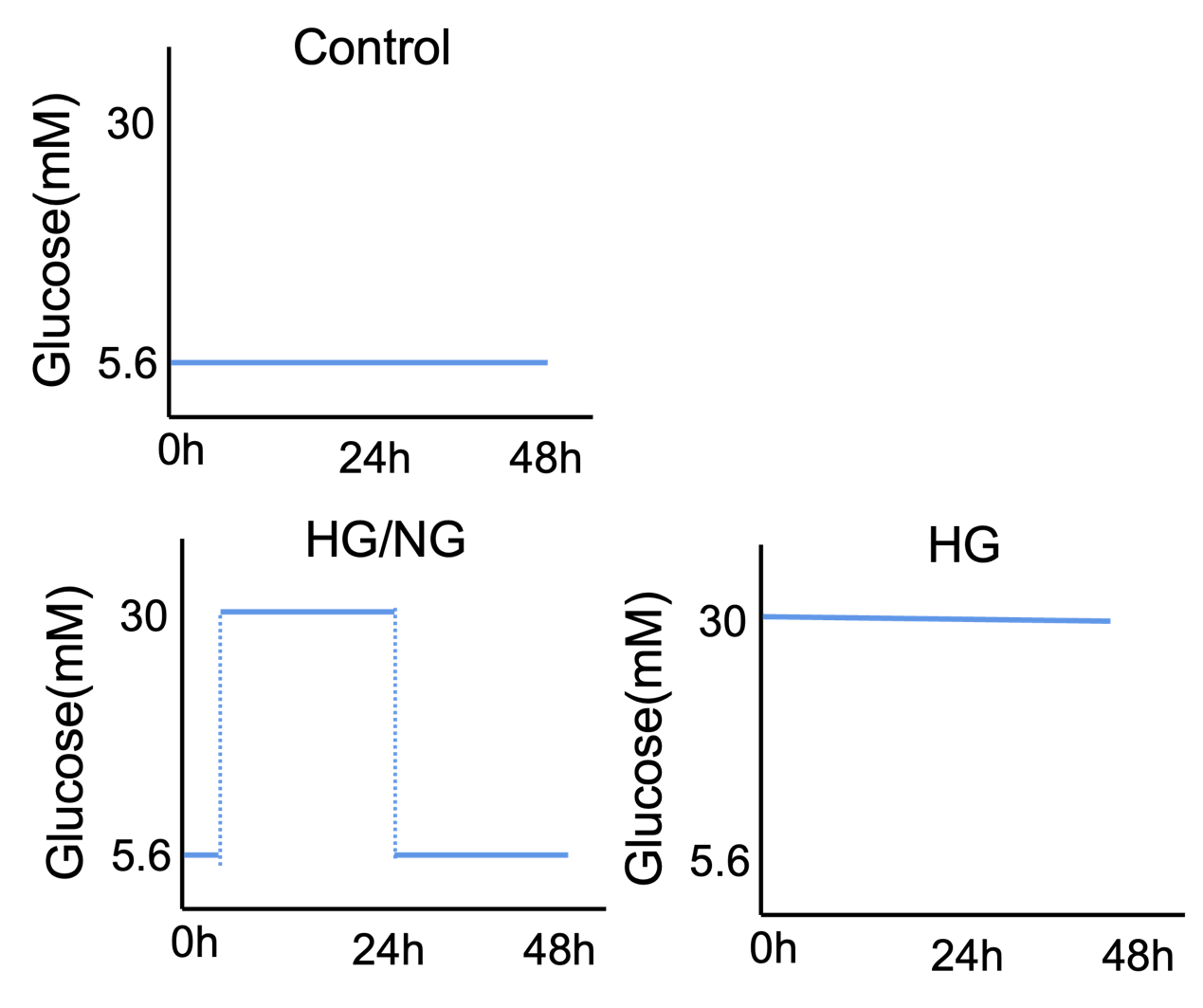
**

Experimental conditions: control with continuously normal glucose (Control, 5 mM glucose), continuously high glucose (HG, 30 mM, 48 h), high glucose for 24 h followed by normal glucose (NG, 5 mM glucose) for 24 h (HG/NG) .

**Supplementary Table 1. Primers for quantitative RT‑PCR**

| Primer | | Sequences |
| --- | --- | --- |
| NDRG1 | Forward | 5’-CATCTCCGCATCGCATCCTCTTC-3’ |
|  | Reverse | 5’-GCTTTGTGAAGTGTGTGCTGCTAC-3’ |
| KCNC4 | Forward | 5’-GAGTTCCTGCTGCTTATCATCTTCC-3’ |
|  | Reverse | 5’-TTCTTGAAGTCGGTGTGGTCATTAC-3’ |
| NR4A1 | Forward | 5’-ATGGACGGCTACACAGGAGAG-3’ |
|  | Reverse | 5’-GGTGGCTGAGGACGAGGATG-3’ |
| ZFP36 | Forward | 5’-GACCTCACCACCACCACCAG-3’ |
|  | Reverse | 5’-GGGCAGCAGAGAAGGCAGAG-3’ |
| β-actin | Forward | 5’-CCTGGCACCCAGCACAAT-3’ |
|  | Reverse | 5’-GGGCCGGACTCGTCATAC-3’ |

**Supplementary Figure 2 Clustering analysis**

Supplementary Figure 2 Clustering analysis. (A) t-distributed stochastic neighbor embedding (tSNE); (B) Principal Component Analysis (PCA); (C) uniform manifold approximation and projection (UMAP).
